# The Emerging Role of *RHOT1*/Miro1 in the Pathogenesis of Parkinson's Disease

**DOI:** 10.3389/fneur.2020.00587

**Published:** 2020-09-15

**Authors:** Dajana Grossmann, Clara Berenguer-Escuder, Axel Chemla, Giuseppe Arena, Rejko Krüger

**Affiliations:** ^1^Luxembourg Centre for Systems Biomedicine (LCSB), University of Luxembourg, Belvaux, Luxembourg; ^2^Section for Translational Neurodegeneration “Albrecht Kossel”, Department of Neurology, Universitätsmedizin Rostock, Rostock, Germany; ^3^Parkinson Research Clinic, Centre Hospitalier de Luxembourg (CHL), Luxembourg, Luxembourg; ^4^Transversal Translational Medicine, Luxembourg Institute of Health (LIH), Strassen, Luxembourg

**Keywords:** Miro1, Parkinson's disease, mitochondrial dynamics, mitophagy, calcium signaling

## Abstract

The expected increase in prevalence of Parkinson's disease (PD) as the most common neurodegenerative movement disorder over the next years underscores the need for a better understanding of the underlying molecular pathogenesis. Here, first insights provided by genetics over the last two decades, such as dysfunction of molecular and organellar quality control, are described. The mechanisms involved relate to impaired intracellular calcium homeostasis and mitochondrial dynamics, which are tightly linked to the cross talk between the endoplasmic reticulum (ER) and mitochondria. A number of proteins related to monogenic forms of PD have been mapped to these pathways, i.e., PINK1, Parkin, LRRK2, and α-synuclein. Recently, Miro1 was identified as an important player, as several studies linked Miro1 to mitochondrial quality control by PINK1/Parkin-mediated mitophagy and mitochondrial transport. Moreover, Miro1 is an important regulator of mitochondria-ER contact sites (MERCs), where it acts as a sensor for cytosolic calcium levels. The involvement of Miro1 in the pathogenesis of PD was recently confirmed by genetic evidence based on the first PD patients with heterozygous mutations in *RHOT1*/Miro1. Patient-based cellular models from *RHOT1*/Miro1 mutation carriers showed impaired calcium homeostasis, structural alterations of MERCs, and increased mitochondrial clearance. To account for the emerging role of Miro1, we present a comprehensive overview focusing on the role of this protein in PD-related neurodegeneration and highlighting new developments in our understanding of Miro1, which provide new avenues for neuroprotective therapies for PD patients.

## Introduction

The mitochondrial Rho GTPase Miro1 was first described in yeast, and these studies already reported a link of Miro1 to calcium homeostasis. Yeast strains devoid of the Miro1-ortholog Gem1p displayed a calcium-dependent growth defect (1). Later, mammalian Miro1 was described as an adaptor for calcium-dependent mitochondrial transport (2–4).

The link between Miro1 dysfunction and Parkinson's disease (PD) arose from studies that identified Miro1 as a target of the PD-associated proteins PINK1 and Parkin. These proteins are mutated in autosomal recessively inherited early-onset PD, and functional studies revealed a key role of PINK1-mediated phosphorylation of Parkin for the regulation of mitophagy as a key mechanism in mitochondrial quality control (5, 6). Therefore, the functional interplay of Miro1 with these key proteins for the maintenance of mitochondrial homeostasis was the first link between mitochondrial dynamics and degradation (7, 8). Further studies *in vivo* revealed that the overexpression of Miro1 in flies led to loss of dopaminergic neurons (9), likely due to a delay of clearance of dysfunctional mitochondria via mitophagy triggered by an excess of Miro1. In contrast, knockout of Miro1 in primary mouse neurons caused a decrease in dendrite complexity as a result of impaired mitochondrial distribution (10). The link between Miro1 dysregulation and neurodegeneration was further substantiated by first studies in human patient-based models showing that impaired Miro1 degradation, and the resulting inhibition of mitophagy, was a shared phenotype in fibroblasts and neurons from different sporadic and monogenic PD patients (11, 12). Recently, our group described the first mitochondria-related cellular phenotypes in fibroblasts from PD patients carrying mutations in *RHOT1*, the gene encoding the Miro1 protein (13, 14), thereby further supporting the involvement of Miro1 in the pathogenesis of PD.

Investigations in yeast showed that Gem1p not only is involved in the regulation of mitochondrial function but also regulates the interplay between mitochondria and the endoplasmic reticulum (ER) (15, 16). This interplay came into the focus of PD research, since several PD-associated proteins were recently identified as regulators of mitochondria-ER contact sites (MERCs), i.e., PINK1, Parkin, LRRK2, or α-synuclein (17–20), all of which are also interacting with Miro1 (7, 11, 12, 21).

Moreover, Miro1 was associated with peroxisomal transport (22–24). Aberrant peroxisome-related metabolism was observed in PD patients (25), and mice with impaired peroxisome activity displayed increased aggregation of α-synuclein (26), providing another potential link between Miro1 and PD via altered peroxisome function. Together, these findings point to an emerging role of Miro1 in neurodegeneration in PD that underscores the need for summarizing the current knowledge about Miro1 and new developments that provide new perspectives for future causative therapies in PD.

## Structure and Physiological Function of The MIRO1 Protein

In mammals, two Miro GTPases, named as Miro1 and Miro2, are encoded by the *RHOT1* and *RHOT2* genes located on chromosome 17. Miro1 and Miro2 are both ubiquitously expressed, consisting of 662 amino acid residues, and display a 60% peptide sequence homology (27–29). Miro GTPases are conserved in almost all eukaryotes containing mitochondria (30), and they were first considered as atypical members of the RAS superfamily of GTPases, particularly as two of the 23 members of the RHO (Ras homolog) protein subfamily (27).

However, in contrast to other RHO family members, Miro GTPases contain no C-terminal cysteine and they also lack the typical RHO insert, which led to their classification as a definite subfamily of small GTPases (29, 31–34).

Structurally distinct N-terminal and C-terminal GTP-binding motifs are present in both Miro proteins, with a linker region (called “MiroS”) connecting two EF-hand domains to the C-terminal GTPase domain (35, 36).

In contrast to the yeast Miro1 homolog Gem1p, which needs both GTPase domains to maintain its function as an adaptor for transport (34, 37), the influence and requirement of these two GTPase domains on mitochondrial trafficking have been widely debated in metazoans, especially in neurons. Several studies suggested that the N-terminal and C-terminal GTPases of Miro1 might have different functions. For instance, it was shown in fly and rat neurons that the C-terminal GTPase domain of Miro is only involved in retrograde transport, while its N-terminal GTPase domain is essential for mitochondrial transport in both retrograde and anterograde directions (2, 38). However, another study provided evidence that alterations of mitochondrial transport in *Drosophila* neurons were exclusively caused by mutations in the N-terminal GTPase domain, but not in the C-terminal GTPase domain of dMiro (*Drosophila* homolog of mammalian Miro1) (37).

Further reinforcing this hypothesis, only mutations in the N-terminal GTPase domain led to the disruption of the mitochondrial network in mammalian cells (28). Moreover, recent work developed by Kalinski et al. describes that the deacetylation of the lysine 105 on the N-terminal GTPase domain of Miro1 could inhibit mitochondrial transport in primary mouse neurons, subsequently affecting axonal growth (39).

While earlier structural studies were performed on dMiro, recent work gave us new insights on human Miro1, demonstrating that both N-terminal and C-terminal GTPases were not only structurally but also functionally different. The N-terminal GTPase was shown to have exclusively GTPase activity, while the C-terminal GTPase also displayed NTPase activity (34, 36, 40), thus making Miro1 the only currently known human protein that contains two different GTPase domains (41). On the other hand, the C-terminal GTPase domain seems to be crucial for the calcium-related functions of Miro1, by interacting with and stabilizing the two EF-hand domains of the protein, which are involved in calcium binding (35, 42).

Miro1 also contains two ligand-mimicking α-helices (LM1 and LM2), which connect each canonical EF hand to a non-canonical “hidden” EF-hand domain (hEF) (35). The calcium-binding amino acids are exposed to the cytosol via an helix-loop-helix-motif in both EF hands, facilitating a conformational change of the protein upon binding to calcium (43, 44).

Moreover, Miro1 harbors a C-terminal transmembrane domain (TMD), which anchors the protein into the outer mitochondrial membrane (OMM), exposing the protein and the N-terminal GTPase to the cytoplasm (2, 35). Fransson et al. demonstrated that the deletion of the TMD in both mammalian Miro proteins led to their mislocalization to the cytoplasm, proving that the TMD is required for mitochondrial targeting (28).

Our group recently described the first heterozygous mutations in the human *RHOT1* gene, found in four individuals diagnosed with PD (13, 14). The identified mutations R272Q, T351A, and R450C were located within highly conserved protein domains of Miro1: R272Q within the LM1 of the N-terminal EF-hand domain, T351A within the C-terminal EF-hand domain, and R450C within the C-terminal GTPase domain (13, 14). The T610A mutation is located within the C-terminus section of the protein, close to the TMD (14). The homology models of the 3D structure of the Miro1 protein showed that all four mutations were localized on the protein surface and exposed to the cytosol. Due to their position, these mutations could therefore impact on calcium binding and sensing, GTP hydrolysis, and mitochondrial localization features of Miro1 (13, 14).

## MIRO1 and Parkinson's Disease

Mitochondria are the main source of cellular energy, and on top of that, they have an essential role in intracellular calcium buffering and regulation of lipid homeostasis (45, 46). For these reasons, dopaminergic neurons critically depend on mitochondrial function, since they require a constant supply of energy and calcium to maintain the integrity of their long axons and to regulate their pacemaking activity for the release and recycling of neurotransmitters (47, 48).

Mitochondrial dyshomeostasis is a central factor in PD pathophysiology, and indeed several genes involved in the development of familial PD are associated with mitochondrial homeostasis (49–51). Increasing evidence indicates that proteins encoded by several PD-linked genes physically interact with Miro1, modifying its function and hence contributing to the dysregulation of neuronal integrity. For this reason, the link between Miro1 and neurodegeneration is a topic of growing interest in PD research.

Bioinformatic analyses indicated that PINK1 and Parkin are direct protein interactors of Miro1 (52). In line with this structural finding, several studies described functional links between these proteins using *in vitro* cellular models from different species. Functional connections between PINK1, Parkin, and Miro1 were first described in flies, where PD-associated deletions in *Drosophila* PINK1 were shown to cause disruption of mitochondrial transport in neuronal axons through interaction with dMiro in a Parkin-dependent manner (9, 53). On top of affecting mitochondrial movement, the loss of PINK1 and Parkin in flies promoted the disruption of other mitochondrial-related mechanisms, such as impairment of mitochondrial clearance, altered the abundance of mitochondria-ER appositions and mitochondrial calcium overload, finally leading to the death of dopaminergic neurons (9, 17). Notably, all these mentioned phenotypes were rescued by a reduction in the amount of dMiro protein in these cells (9, 17), emphasizing the importance of the multifunctional role of Miro1 for mitochondrial homeostasis in PD.

Despite the clear link between mitochondrial dyshomeostasis in PD and Miro proteins, single-nucleotide polymorphisms (SNPs) in *RHOT1/2* were not associated with PD using genome-wide association studies (GWAS) (54), and recent meta-analyses of GWAS data did not identified *RHOT1/2* as risk loci for PD (55, 56). However, GWAS are not designed to detect rare variants due to a minor allele frequency of the used SNPs of >5% in most studies, and therefore, sequencing methods to identify rare variants are needed. Of note, gene-based association clustering methods recently allowed the identification of *RHOT2*, the gene encoding for Miro2, as a PD-associated gene (57). Recently, our group identified the first heterozygous mutations in the *RHOT1* gene in four independent PD patients by exome sequencing (13, 14), further strengthening the impact of Miro1 in the development of PD and defining *RHOT1* as a potential novel risk gene for the pathogenesis of this disorder.

The knowledge about the role of human Miro1 in neurodegeneration, particularly in the pathogenesis of PD, is growing rapidly. In the next sections, we will discuss the molecular and cellular effects of mutant human Miro1 in PD.

## MIRO1 as a Target For Pink1/Parkin-Mediated Mitophagy

An impressive number of studies over the last 30 years shed light on the so-called mitochondrial life cycle, during which these highly dynamic organelles continuously experience fission and fusion events to meet the functional needs of the cells. Maintaining this mitochondrial network requires the coordinated activity of mitochondrial biogenesis and clearance pathways, which ensure the replacement of damaged organelles with metabolically active mitochondria. Consequently, impairing this fine-tuned quality control mechanism leads to the accumulation of dysfunctional mitochondria, which in turn increases oxidative stress and deteriorates cellular activity (58, 59). Removal of damaged mitochondria is even more important in post-mitotic cells like neurons, which are not able to dilute harmful components through cell division.

In accordance, mitochondrial dysfunction plays an essential role in a large number of neurodegenerative diseases, including PD, and the accurate mitochondrial turnover is now considered a key neuroprotective mechanism against chronic disease conditions (60).

Mitochondrial degradation is a well-orchestrated process involving the two main intracellular clearance machineries, namely, the ubiquitin–proteasome system (UPS) and the autophagy pathway (61). The PD-linked PINK1 and Parkin proteins are the master and commander of this multistep mechanism: (i) the mitochondrial kinase PINK1 selectively recognizes depolarized mitochondria and rapidly accumulates on their surface, where it starts a massive phosphorylation of ubiquitinated proteins; (ii) the cytosolic ubiquitin-ligase Parkin recognizes PINK1-catalyzed phospho-ubiquitin and translocates to mitochondria, supplying further ubiquitin chains to PINK1 and amplifying the signal in a positive feedback loop; (iii) Parkin ubiquitinates a number of substrates on the OMM, leading to the inhibition of mitochondrial fusion and arrest of mitochondrial movement; (iv) the coating of dysfunctional mitochondria with phospho-ubiquitin chains recruits specific components of the autophagic receptor machinery to mitochondria, which are then engulfed by autophagosomes; and (v) finally degraded into lysosomes (62).

**Figure 1:**
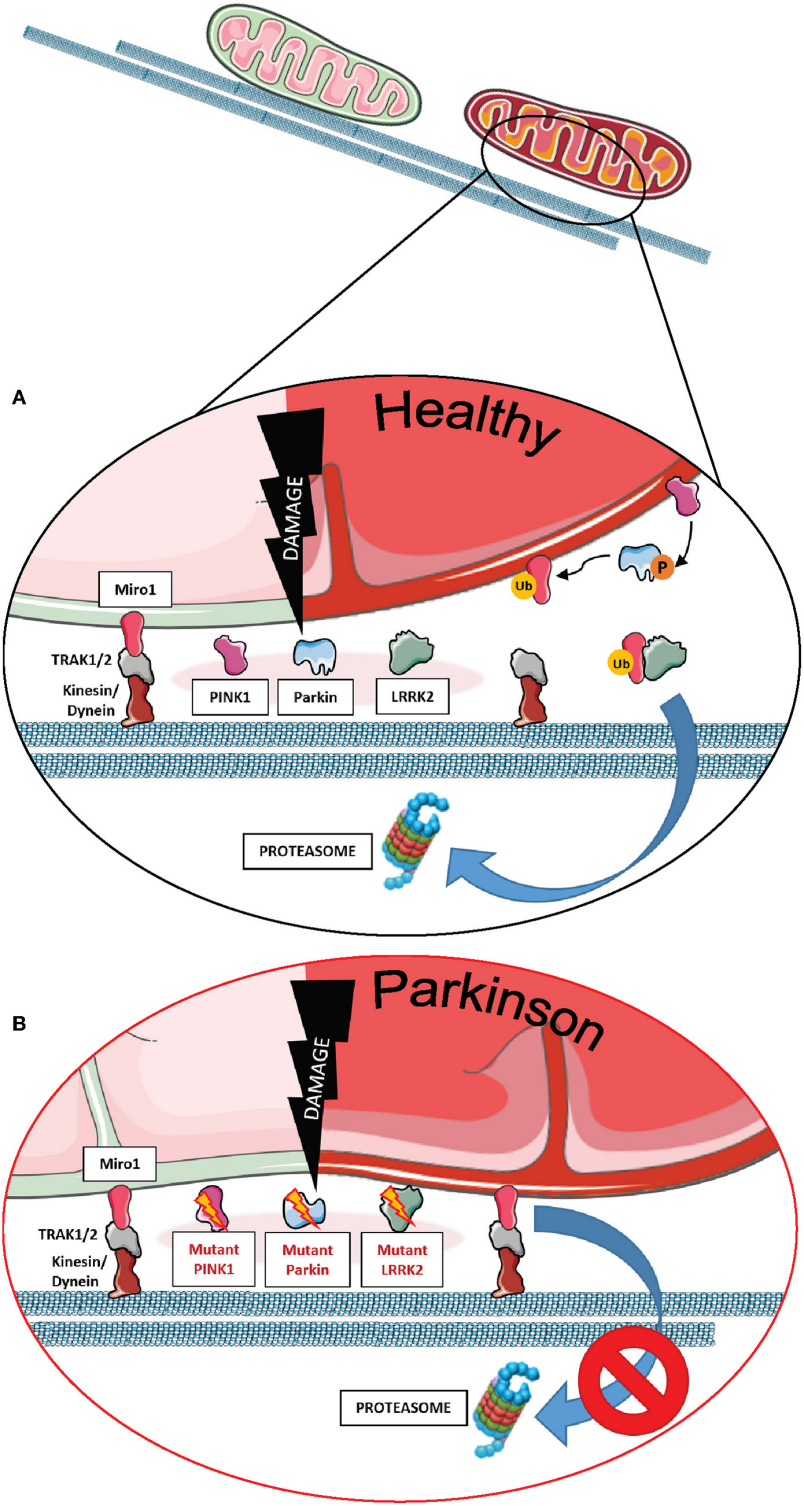
The role of Miro1 in mitophagy. **(A)** Lysosomal degradation of dysfunctional mitochondria requires the stop of mitochondrial transport and detachment from the cytoskeleton. Mitochondrial damage leads to the accumulation of PINK1 at mitochondria and the recruitment of the E3 ubiquitin ligase Parkin. PINK1 phosphorylates and activates Parkin, which in turn ubiquitinates proteins at the outer mitochondrial membrane, including Miro1. Additionally, PINK1 might also phosphorylate ubiquitin chains on mitochondrial proteins. Ubiquitinated proteins, including Miro1, are then targeted for proteasomal degradation, thereby disconnecting mitochondria from the cytoskeleton and stopping transport. Isolated mitochondria are then ready for uptake by autophagosomes. LRRK2 was shown to be involved in the removal of Miro1 from the surface of impaired mitochondria. **(B)** In cell models expressing PD-associated mutations in PINK1, Parkin, or LRRK2, the proteasomal degradation of Miro1 is impaired, consequently interfering with the arrest of mitochondrial transport and the initiation of mitophagy. This figure was created using elements from Servier Medical Art, licensed under a Creative Common Attribution 3.0 Generic License (www.smart.servier.com).

Miro1 plays an essential role in this process, being one of the first substrates of Parkin E3-ligase activity. As a key component of the mitochondrial transport machinery that anchors the organelle to the motor proteins of the cytoskeleton, Miro1 is ubiquitinated by Parkin and consequently degraded by the UPS (Figure 1A), which leads to mitochondrial arrest and facilitates mitophagy (7–9, 36, 42, 63, 64). Particularly in the context of PD, PD-associated mutations in Parkin were shown to disrupt the ubiquitination of Miro1 for proteasomal degradation in patient-derived fibroblasts, leading to the inhibition of Miro1 turnover and the subsequent failure of mitochondrial arrest for mitophagy (Figure 1B) (7). Further supporting the functional interaction of Miro1 with other PD gene products linked to mitochondrial quality control, previous findings also showed that Miro1 physically interacts with PINK1 and is phosphorylated by the kinase on the serine 156, which could represent a signal for the following ubiquitination by Parkin (7, 65).

In addition to the PINK1-Parkin axis, Miro1 removal from the OMM of depolarized organelles could also be mediated by its association with other PD-related proteins (Figures 1A,B). Two studies supported this hypothesis by the discovery that LRRK2 and α-synuclein cooperate with Miro1 to stop mitochondrial movement prior to mitophagy (11, 12). In fact, the pathogenic PD mutations LRRK2 G2019S and α-synuclein A53T disrupt this process, resulting in Miro1 accumulation, delayed mitochondria arrest, and impaired mitophagy activation in patient-derived fibroblasts and induced pluripotent stem cell (iPSC)-derived neurons (11, 12).

Based on these findings, clearing Miro1 from depolarized mitochondria is emerging as a potential neuroprotective mechanism against PD, as the failure of its removal from mitochondria was demonstrated in cells from PD patients. Interestingly, in some PD cases, Miro1 degradation is impaired even in the presence of functional Parkin and LRRK2, indicating the existence of additional mechanisms accounting for Miro1 removal from dysfunctional mitochondria (66). Hence, Hsieh et al. demonstrated that genetically or pharmacologically reducing Miro1 levels improved mitochondrial arrest, activated mitophagy, and prevented dopaminergic neurodegeneration in both iPSC-derived human neurons and fly models of PD, without significantly affecting the movement of healthy mitochondria (11, 12, 66).

Further confirming an important role for Miro1 in the pathogenesis of PD, we recently described mitophagy alterations in fibroblasts from PD patients harboring heterozygous mutations in the *RHOT1* gene encoding Miro1 (13, 14). Interestingly, all mutant fibroblast lines demonstrated alterations in mitophagy flux, but the resulting phenotype was different depending on the Miro1 mutation. In fact, the R272Q and R450C mutants demonstrated increased levels of mitophagy compared to controls, reflected by increased mitochondria co-localizing with LC3 puncta and decreased Parkin protein levels under baseline conditions. CCCP treatment was not sufficient to further increase mitophagy, suggesting that mitophagy was already running at maximal capacity (13). In contrast, the T351A and T610A mutants displayed no increase in mitophagy under baseline conditions. CCCP treatment leads to increased co-localization of LC3 puncta with mitochondria in control cells, but not in Miro1-T351A or -T610A fibroblasts, suggesting an impaired mitophagy mechanism in these mutants (14).

It is worth noting that, in contrast to the increased mitophagic turnover observed in R272Q mutant fibroblasts, iPSC-derived neurons harboring the same Miro1 mutation displayed an opposite phenotype compared to the fibroblasts (67). Mitophagy was not inducible in Miro1-R272Q neurons, either by oxidative stress or by CCCP treatment. Furthermore, bafilomycin A1 treatment did not lead to an accumulation of the autophagic cargo protein p62 in these cells, suggesting a reduced autophagic turnover (68). Based on these observations, mitophagy seems to be regulated differentially in fibroblasts and neurons.

**Figure 2:**
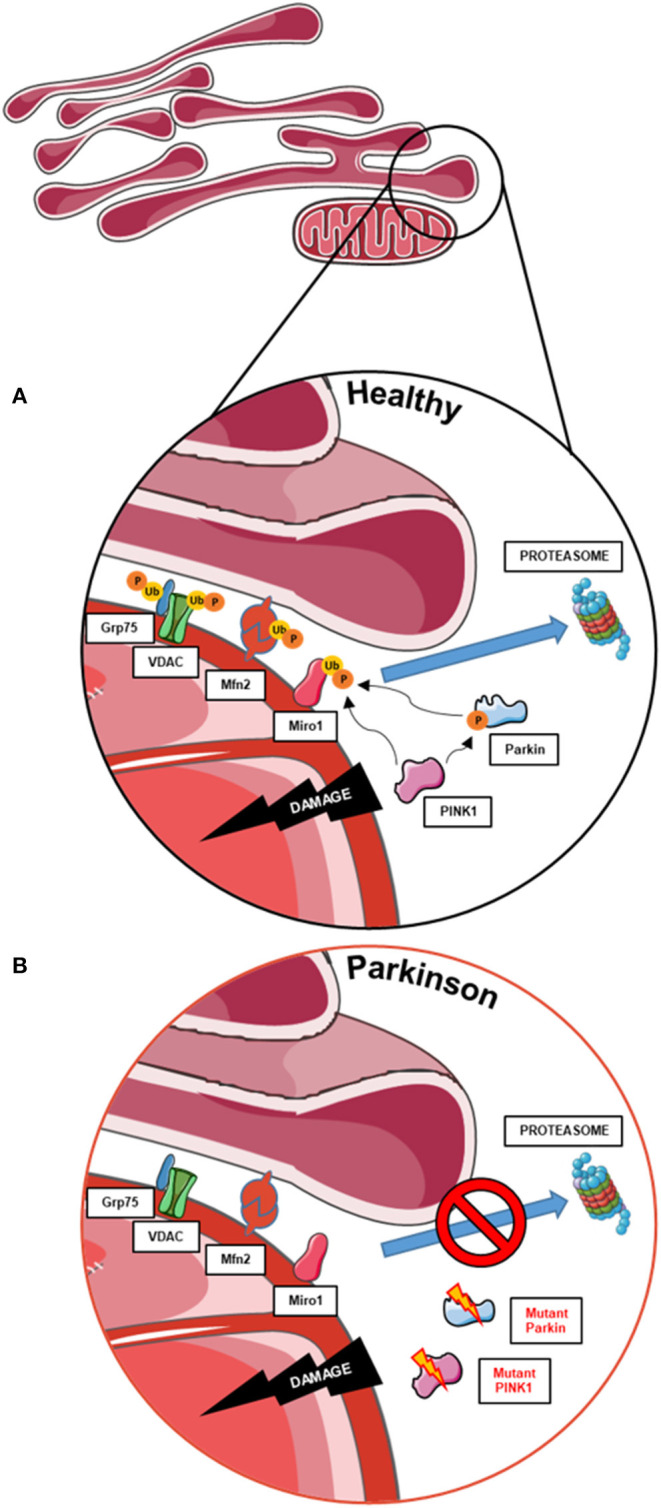
The role of MERCs in mitophagy and the contribution of PD-associated proteins. **(A)** Mitophagy also requires the untethering of impaired mitochondria from the ER. Therefore, PINK1 and Parkin work together to ubiquitinate and phosphorylate proteins involved in subtypes of MERCs (i.e., Grp75, VDAC, Mfn2, and Miro1) for subsequent proteasomal degradation and disassembly of MERCs, allowing the degradation of impaired mitochondria. **(B)** In cells with impaired PINK1 or Parkin function, MERCs are not disassembled upon mitochondrial dysfunction, hence hampering the initiation of mitophagy. This figure was created using elements from Servier Medical Art, licensed under a Creative Common Attribution 3.0 Generic License (www.smart.servier.com).

Remarkably, the mitophagy phenotype in these cells seems to be tightly related to the degree of topographic association between mitochondria and ER, as represented by the distance between both organelles (68). In 2018, McLelland et al. described that the initiation step of mitophagy in human cancer cell lines and iPSC-derived neurons occurs at mitochondria and ER appositions where the cleft that separates both organelles is wider than ~30 nm (Figure 2A) (69). These “wide” appositions serve as a platform for Parkin-mediated ubiquitination of OMM proteins at depolarized mitochondria, subsequently promoting the uncoupling of mitochondria from the ER and the mitophagy process (13, 14). This hypothesis was drawn from the observation that iPSC-derived neurons from a PD patient with a deletion in Parkin did not show a decrease of these “wide” MERCs after initiation of mitophagy with CCCP (Figure 2B) (69). Fitting, our results showed that Miro1-mutant lines with an unchanged number of “wide” MERCs compared to controls displayed the ability to induce mitophagy after CCCP treatment (13, 14), while mutants with reduced amount of “wide” MERCs revealed a deficit to initiate mitophagy after treatment with CCCP (14). These findings suggest that the regulation of mitochondrial quality control by Miro1 might crucially depend on the structure of MERCs.

## MIRO1 as a Regulator of Mitochondrial-ER Contact Sites

MERCs are discrete areas of proximity between mitochondria and the ER that coordinate essential physiological processes, such as lipid biosynthesis, cellular calcium handling, and mitochondrial homeostasis (70–72). These mechanisms are reported to be affected in neurodegeneration (73, 74); hence, MERCs are one of the most studied organelle juxtapositions and a current spotlight in PD research (75).

Several mitochondria-related proteins involved in PD pathogenesis modulate the physiological function of the MERCs by acting as regulatory factors. Overexpression of Parkin in HeLa cells was shown to increase physical and functional coupling between mitochondria and ER, stimulating mitochondrial calcium uptake and ATP production, while Parkin knockdown had the opposite effect (18). Similarly, overexpression of α-synuclein and DJ-1 proteins increased the number of MERCs in HeLa cells, subsequently increasing mitochondrial calcium uptake (18, 76). LRRK2 was recently found to also modulate MERC amount and function, since LRRK2-null MEFs express reduced MERC abundance and dysregulated mitochondrial calcium uptake (77). Moreover, α-synuclein was found in MERCs from mouse and human brain tissue, where it seems to modulate mitochondrial morphology (78). Like α-synuclein, PINK1 was recently found to also localize to MERCs, and its continuous degradation in healthy mitochondria is regulated by the interplay of mitochondria and the ER (79). It is worth noting that the PD-related proteins PINK1, Parkin, LRRK2, and α-synuclein that are involved in MERCs have also been shown to directly or indirectly interact with Miro1 (7, 11, 12, 21). Hence, impaired mitophagy and dysregulation of MERCs seems a shared feature in different cases of PD.

The relationship between Miro proteins and MERCs started to be investigated when, in 2011, two research groups identified Gem1p, the yeast ortholog of mammalian Miro1, as a crucial regulator of the ER-mitochondrial encounter structure (ERMES), a protein complex that tethers mitochondria and ER in yeast (15, 80). Association of Gem1p to ERMES controls phospholipid exchange for lipid biosynthesis between mitochondria and ER (15) and regulates mitochondrial division and morphology (81–83). The localization of dMiro at MERCs was also demonstrated in *Drosophila* neural stem cells and dopaminergic neurons (17, 84), as well as mammalian Miro1 in COS-7 cells, HeLa cells, MEFs, human fibroblasts, and human iPSC-derived neurons (14, 15, 67, 84, 85).

**Figure 3:**
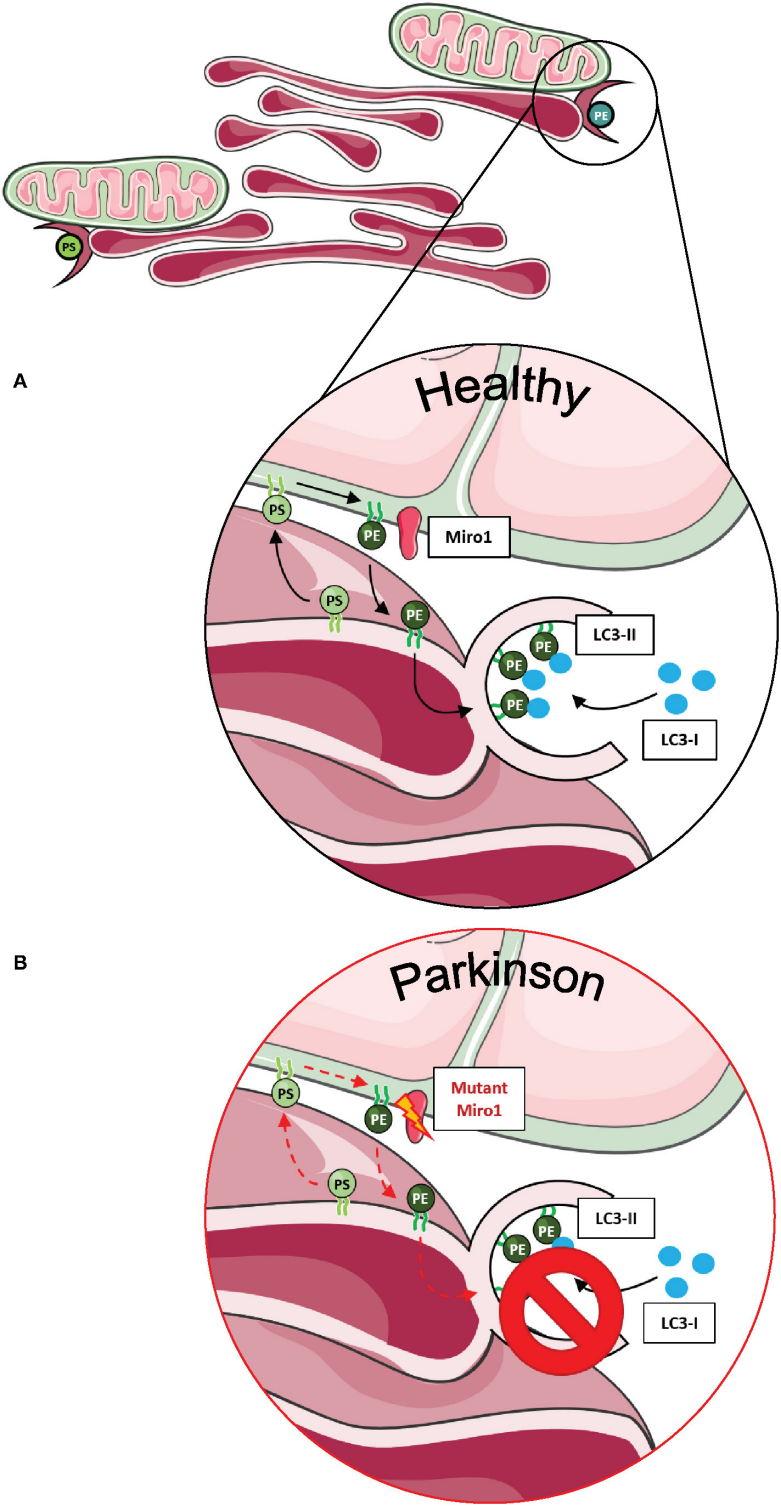
Phospholipid synthesis and autophagosome formation at MERCs. **(A)** Phospholipids are synthesized in mitochondria and the ER, requiring the exchange of metabolites at MERCs. Phosphatidylserine (PS) is synthesized in the ER, shuttled to mitochondria via MERCs, where it is transferred into phosphatidylethanolamine (PE) and shuttled back into the ER. PE is necessary for the assembly of isolation membranes at the ER for the integration of cytosolic LC3-I into the autophagosome membrane, forming LC3-II. **(B)** A number of studies suggest that Miro1 is involved in phospholipid shuttling via MERCs. Disruption of Miro1 function might impair phospholipid synthesis, consequently interfering with the formation of autophagosomes. This figure was created using elements from Servier Medical Art, licensed under a Creative Common Attribution 3.0 Generic License (www.smart.servier.com).

In human-derived cells, the contribution of Miro1 in lipid exchange and biosynthesis was confirmed by the discovery that patient-derived fibroblasts with PD-associated mutations in Miro1 displayed an altered formation of autophagosomes, which is dependent on the conversion of phosphatidylserine (PS) to phosphatidylethanolamine (PE) at MERCs (13, 14). In mammalian cells, PS is synthesized in the ER, transferred through the MERCs to the mitochondria, and transformed into PE (72). A fraction of the mitochondrial-generated PE is then shuttled back to the ER for the generation of isolation membranes, where PE is used for the lipidation of specific adaptor proteins that recruit autophagic cargoes (Figure 3A) (86, 87). In our studies, none of the Miro1-mutant fibroblast lines showed an increase in the amount of newly synthesized autophagosomes following starvation conditions (13, 14). In line with these results, all Miro1-mutant fibroblast lines showed an overall reduction in MERCs, suggesting that PD-associated Miro1 mutations disturb the formation of appositions between mitochondria and ER, affecting lipid exchange and, consequently, autophagy initiation (Figure 3B) (13, 14).

In contrast to our observations in patient-derived fibroblasts, Miro1-R272Q neurons showed an increased number of contacts between mitochondria and ER (67). Remarkably, only control neurons exhibited accumulation of the autophagic cargo protein p62 upon bafilomycin A1 treatment, but not Miro1-R272Q neurons (67). These results point toward a functional impairment of MERCs that may affect the initiation of autophagy in patient-derived neurons, possibly triggered by the pathogenic effect of mutant Miro1.

**Figure 4:**
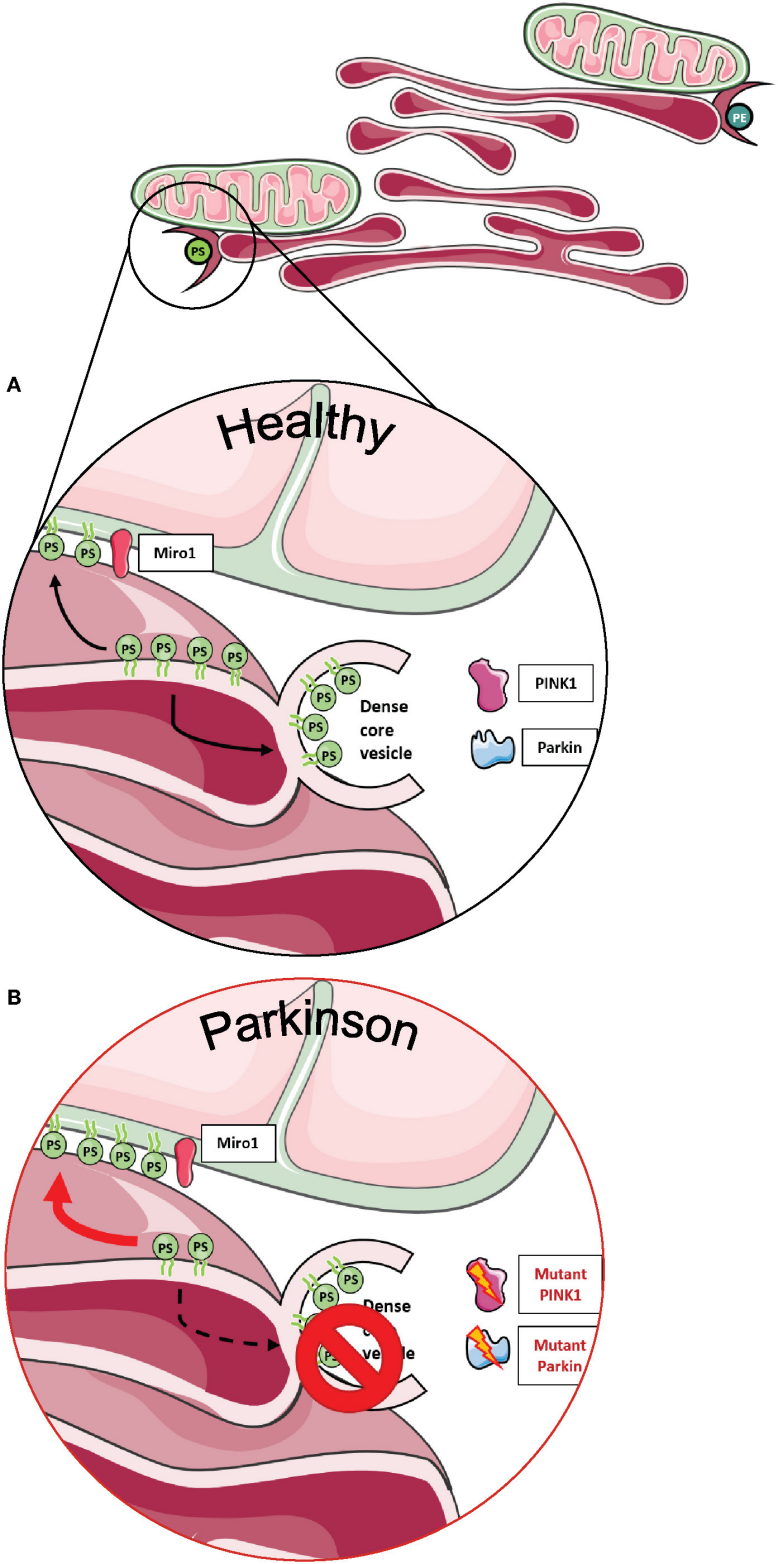
Phospholipid synthesis and the formation of dense core vesicles at MERCs. **(A)** Phospholipids are required not only for the formation of autophagosomes, but PS is also required to provide membranes for dense core vesicles. **(B)** Fly neurons expressing mutant PINK1 or Parkin show impaired formation of dense core vesicles, resulting in alterations of neurotransmission. This figure was created using elements from Servier Medical Art, licensed under a Creative Common Attribution 3.0 Generic License (www.smart.servier.com).

Importantly, two Miro1 interactor proteins, PINK1 and Parkin, were also shown to be involved in the organization and lipid-related function of MERCs (Figure 4A). Neurons derived from flies and patients carrying mutations in PINK1 and Parkin displayed increased amounts of MERCs and a disturbed exchange of the phospholipid PS, resulting in an impaired synthesis of dense core vesicles from the ER (Figure 4B) (88). Altogether, these findings argue in favor of an important role of Miro1 in lipid homeostasis at MERCs and an impairment of this function in conditions linked to neurodegeneration.

Other studies in metazoans also supported a key role of Miro in the regulation of MERCs. In *Drosophila* neural-derived cultures, Polo kinase-induced phosphorylation of dMiro enhances the localization of dMiro to MERCS and the interaction with calcium transporters to regulate calcium homeostasis and the integrity of the tethering complex (17, 84). Moreover, in our studies, we were able to observe a reduced co-localization of Miro1 with MERCs in Miro1-mutant fibroblasts from PD patients compared to control fibroblasts (14) and, conversely, an increased co-localization of Miro1 with MERCs in Miro1-R272Q iPSC-derived neurons (67), underscoring the importance of Miro1 localization to MERCs and a potential role in neurodegeneration.

As mentioned in the previous section, MERCs were recently shown to act as regulators of mitophagy initiation (Figure 2A). Coupled mitochondria and ER in human iPSC-derived dopaminergic neurons are untethered upon Parkin-mediated ubiquitination of MERC-residing proteins, such as Mfn2 and VDAC (63, 89, 90), as a starting point for mitochondrial clearance (69). Based on the evidence that targeting of Miro1 by the PINK1/Parkin pathway is required as an initial step for mitophagy (7, 9, 91), these studies provide strong evidence that PINK1/Parkin-mediated mitophagy is organized at MERCs and that Miro1 might be directly involved in that process. Indeed, fibroblasts obtained from PD patients harboring Miro1 mutations show significant alterations in mitophagy flux accompanied by dysregulation of the abundance of specific subtypes of MERCs, supporting the previous hypothesis (13, 14).

Moreover, based on the increased amount of overall MERCs and impaired CCCP-induced mitochondrial clearance observed in iPSC-derived Miro1-R272Q neurons, we speculate that damaged mitochondria may not uncouple from the ER, consequently hampering the initiation and flux of mitophagy (67).

In conclusion, the communication between mitochondria and ER is crucial to maintaining cellular homeostasis and is a potential investigation target of growing interest in neurodegenerative diseases, such as PD. Miro1 was demonstrated to be crucially involved in the regulation of the function of the MERCs; therefore, the study of this interaction between Miro1, mitochondria, and ER will help to better comprehend the complex pathogenicity of PD.

## MIRO1 as a Regulator of Cellular Calcium Homeostasis

Calcium ions act as important second messengers that control several cellular mechanisms. Therefore, cytosolic calcium levels need to be tightly regulated, and cells manage to maintain calcium homeostasis mainly via buffering calcium by specific organelles, such as the ER and mitochondria (84, 92).

One of the main functions of Miro1 is to orchestrate calcium homeostasis in mitochondria and calcium-dependent mitochondrial positioning. To fulfill this function, calcium-binding is facilitated via both of its EF-hand domains (33, 93) and the C-terminal GTPase domain (35, 42). Miro1 was suggested to have a high calcium-binding affinity (33) and consequently binds calcium only upon elevation of cytosolic calcium levels (3, 42, 94).

Interestingly, Chang et al. found in 2011 that intra-mitochondrial calcium levels correlated with mitochondrial transport speed, suggesting that mitochondrial transport was not only controlled by cytosolic calcium transients but also by mitochondrial matrix calcium levels (4). Furthermore, primary mouse neurons overexpressing Miro1 with calcium-insensitive EF-hand domains showed a decreased influx of calcium into the mitochondrial matrix, suggesting that Miro1 also regulates intra-mitochondrial calcium levels (4).

**Figure 5:**
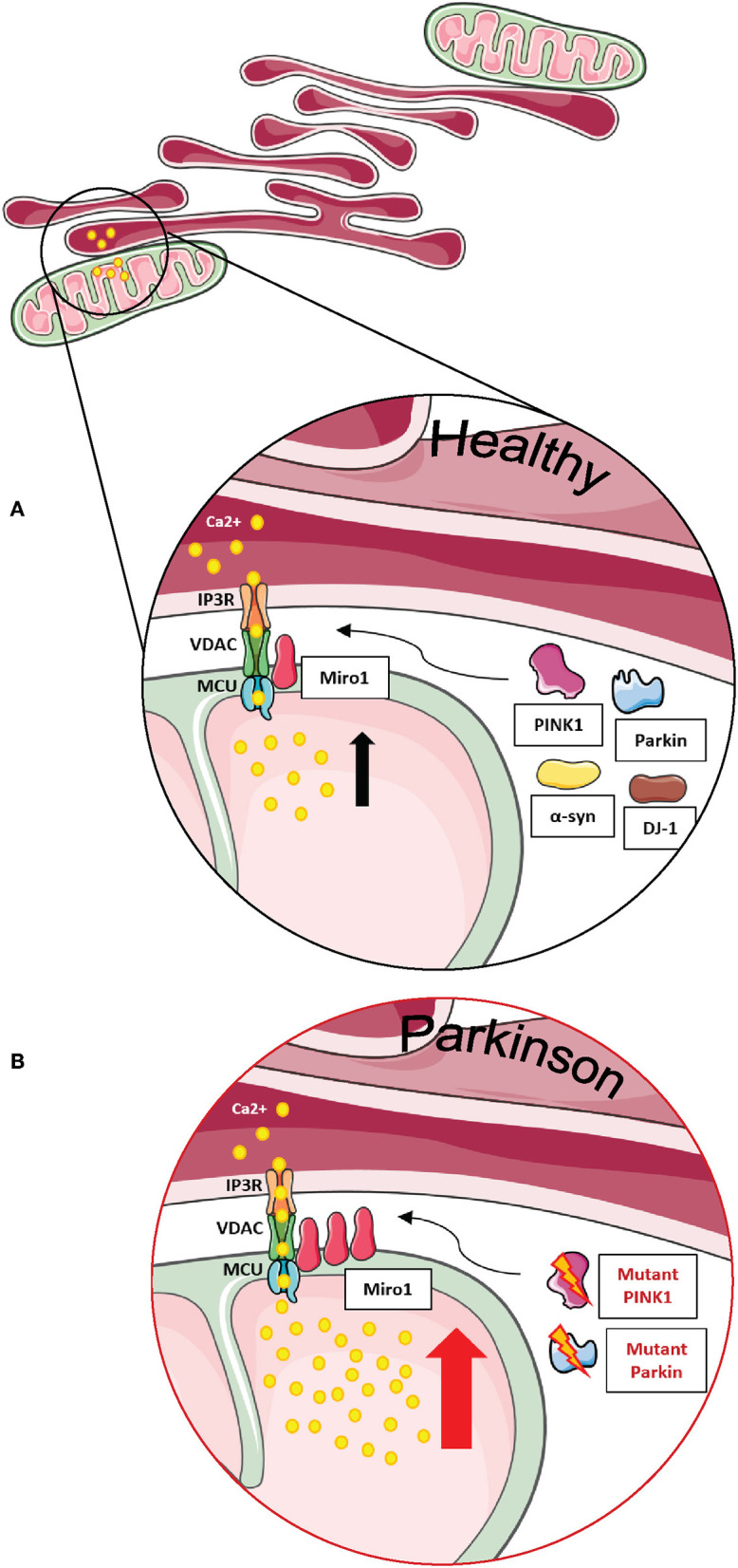
Miro1 is involved in calcium regulation at MERCs. **(A)** Calcium homeostasis is tightly regulated. ER and mitochondria buffer cytosolic calcium transients and specialized subtypes of MERCs composed of IP3R, VDAC, MCU, and Miro1 are required for regulation of calcium uptake. Miro1 acts as sensor of cytosolic calcium levels, interacting directly with the MCU and orchestrating the mitochondrial calcium uptake at MERCs. PD-associated proteins PINK1, Parkin, α-synuclein, and DJ-1 participate in the regulation of calcium homeostasis at MERCs. **(B)** Impaired PINK1 or Parkin function leads to clustering of Miro1 and subsequent mitochondrial calcium overload that facilitates mitochondrial dysfunction and apoptosis. Additionally, impaired Miro1 function leads to disruption of cellular calcium homeostasis by alterations of mitochondrial calcium buffering. This figure was created using elements from Servier Medical Art, licensed under a Creative Common Attribution 3.0 Generic License (www.smart.servier.com).

This finding was later supported by a study in primary fly neurons. Knockdown of dMiro caused a decreased histamine-induced calcium uptake in the mitochondrial matrix, while overexpression of dMiro led to increased calcium uptake (84). Lee et al. concluded that dMiro specifically promotes the flux of calcium from the ER to mitochondria and that this mechanism is independent of mitochondrial transport and intracellular distribution (Figure 5A) (84).

While the effect of intra-mitochondrial calcium levels on mitochondrial transport seems surprising at first glance, it is known that increased cytosolic calcium transients lead to an unavoidable influx of calcium into the mitochondrial matrix (4). The observed regulation of mitochondrial matrix calcium by Miro1 (4, 84) raised the question how a protein bound to the OMM and facing the cytosol can possibly regulate the influx of calcium into the matrix.

Uptake of calcium into mitochondria is facilitated by the mitochondrial calcium uniporter (MCU), a protein complex of several subunits residing in the inner mitochondrial membrane. Only in 2018, Niescier et al. revealed that the N-terminus of the MCU reaches through the mitochondrial intermembrane space and the outer membrane to directly interact with Miro1 (Figures 5A,B). This study finally solved the question how Miro1 residing in the outer membrane is able to regulate intra-mitochondrial calcium homeostasis (95).

Regulation of mitochondrial matrix calcium levels is important for mitochondrial energy production (96–98). Hence, loss-of-function mutations in Miro1 were suggested to affect mitochondrial energy production by disrupting the mitochondrial matrix calcium uptake (86). Indeed, the ATP production was decreased in brains of *Drosophila* larvae expressing the loss-of-function mutation dMiro B682 (84), in GemA (ortholog of Miro) knockout *Dictyostelium discoideum* (30), and in fibroblasts from patients carrying PD-associated Miro1 mutations (13). Together, these findings suggest that Miro1 plays a crucial role in the maintenance of mitochondrial function via regulation of mitochondrial calcium levels.

The importance of Miro1 for the maintenance of calcium homeostasis in the context of PD was highlighted in our recently published studies with PD patient-derived fibroblasts (13, 14). In these studies, we used thapsigargin, an inhibitor of the sarco-/ER calcium ATPase (99). When the ER calcium uptake was blocked by thapsigargin treatment: (i) calcium levels in the cytosol rose rapidly due to depletion of the ER calcium store (100), and (ii) calcium buffering relied on other mechanisms, i.e., mitochondrial calcium uptake. We found that buffering of cytosolic calcium after thapsigargin treatment was delayed in patient-derived fibroblasts harboring mutations in Miro1 (13, 14), suggesting that mitochondrial calcium buffering is impaired in Miro1-mutant fibroblasts. In addition, combined treatment of thapsigargin with the MCU inhibitor Ru360 (4, 101, 102) caused a reduction in cytosolic calcium buffering in the control fibroblast lines similarly to Miro1-mutant fibroblasts. These results confirmed that calcium buffering relies mostly on mitochondria when calcium uptake via the ER is blocked (13, 14).

Our studies also supported previous observations where mutations in the EF-hand domains of Miro1 caused an elevation of the frequency of calcium spikes and an increase in the time constant of calcium transients in primary rat astrocytes (103). A similar disruption of calcium homeostasis with increased frequency and amplitudes of calcium spikes was observed in rat hippocampal cultures with deletion of Miro1 EF-hand domains (Miro1-ΔEF) (104). In contrast, overexpression of wild-type Miro1 led to decreased thapsigargin-induced calcium spikes in primary fly neuron cultures (84).

Maintenance of calcium transients is important for the function of astrocytes and neurons. High levels of calcium enter the cell at active synapses and need to be buffered via mitochondria (103, 104). Impaired cellular calcium homeostasis is a shared phenotype observed in different models of PD (105–110). In line with this, iPSC-derived neurons with heterozygous Miro1-R272Q display a significantly higher peak of cytosolic calcium and delayed buffering capacity after ionomycin treatment compared to control neurons (67). Hence, one might speculate that mutations in Miro1 drive neurodegeneration by impairing calcium homeostasis, subsequently affecting mitochondrial function and energy production in the pathogenesis of PD. Indeed, *Drosophila* expressing loss-of-function mutations in the EF-hand domains of dMiro show a decreased neuronal survival because the impaired Miro-mediated calcium-dependent mitochondrial positioning affects calcium homeostasis and thereby increasing the susceptibility to glutamate excitotoxicity (3).

Another function of Miro1 is the regulation of mitochondrial dynamics in a calcium-dependent fashion. Glutamate application to primary rat neuronal cultures caused a reduction in mitochondrial length. However, these calcium-dependent changes in mitochondrial morphology were abolished in cells expressing Miro1-ΔEF (104). In 2018, Nemani et al. revealed that this Miro1-mediated calcium-dependent mitochondrial fragmentation was independent of the mitochondrial fission protein Drp1, the mitochondrial membrane potential, or the production of reactive oxygen species (ROS) and is an important prerequisite of mitophagy (94).

In our studies, PD patient-derived Miro1-mutant fibroblasts and iPSC-derived Miro1-R272Q neurons demonstrated an increased mitochondrial fragmentation after treatment compared to control cells. This finding was not surprising given the delayed buffering of calcium transients and resulting retained high levels of calcium in the cytosol (13, 14, 67). Our findings imply that mutations in Miro1 cause an impairment of calcium homeostasis, resulting in decreased ATP production and increased calcium-dependent mitochondrial fragmentation, thereby contributing to the pathogenesis of PD.

## MIRO1 and Organellar Movement In Parkinson's Disease

### Mitochondrial Transport

Miro1 is a well-known adaptor for the mitochondrial transport machinery, forming a complex with the motor proteins dynein, kinesin, and myosin and thereby allowing mitochondrial movement along the cytoskeleton (2, 28, 37, 111, 112).

This function of Miro1 is especially crucial in neurons, as anterograde mitochondrial transport (from soma to synapses) is necessary to provide ATP and ensure calcium buffering at highly energy-demanding areas, such as synapses (113). An appropriate mitochondrial distribution is even more essential in dopaminergic neurons, because their pacemaking activity requires high-energy supply and makes them more vulnerable to excitotoxicity (113).

Retrograde mitochondrial movement is as well essential for the physiology of neurons *in vivo*, since lysosomal degradation of damaged mitochondria takes place mostly in the neuronal soma (113, 114).

Guo et al. first demonstrated the importance of dMiro for anterograde mitochondrial transport in neurons from *Drosophila* larvae (37). However, other studies reported that the expression of the null alleles B682 and SD32 within the first GTPase domain of dMiro, which cause the loss of the protein through its premature truncation, promoted a reduction in both anterograde and retrograde transport of mitochondria (111). Interestingly, the N-terminal GTPase domain of dMiro seems to be crucial for mitochondrial transport along axons and dendrites (38). In accordance, expression of the N-terminal GTPase loss-of-function mutation dMiroT25N led to premature death and aborted development of *Drosophila puparium*, a phenotype likely due to an accumulation of dysfunctional fragmented mitochondria in the soma of their sensory and motor neurons (38). Recently, inhibition of mitochondrial transport was linked to HDAC6-mediated deacetylation of the N-terminal GTPase domain of Miro1 in rodents (39). In addition, Miro1 knockout mice displayed a reduced number of mitochondria in distal dendrites, accompanied by a lower dendritic complexity and increased neuronal death (10). Of note, these mice are still expressing Miro2, suggesting that although both isoforms (Miro1 and Miro2) are involved in mitochondrial transport, they are not able to fully substitute each other (10).

**Figure 6:**
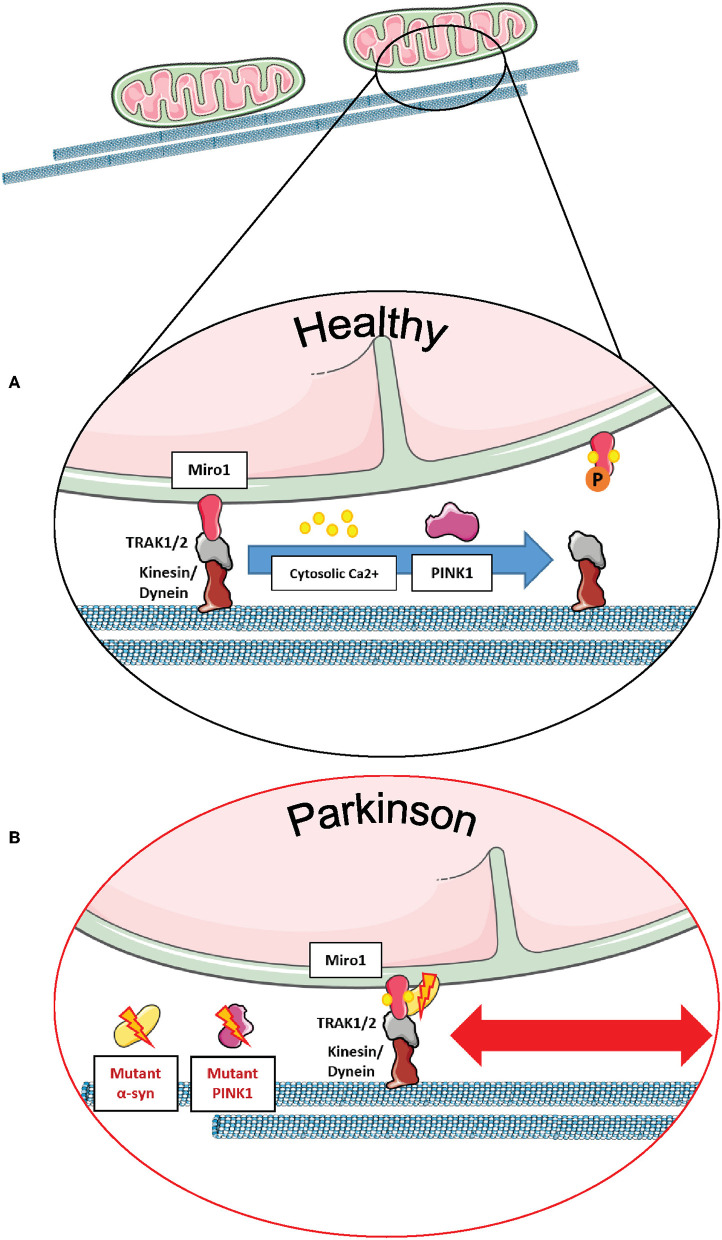
Miro1 mediates calcium-dependent regulation of mitochondrial transport. **(A)** Miro1 is anchored to the outer mitochondrial membrane and links mitochondria to the motor proteins kinesin and dynein via interaction with TRAK1/2. Upon elevation of cytosolic calcium levels (i.e., at active synapses), calcium binds to the EF-hand domains of Miro1, leading to a conformational shift of Miro1 and a decoupling of mitochondria from the cytoskeleton. Thus, mitochondria are stopped at sites of high calcium levels, providing ATP and calcium buffering. PINK1/Parkin-mediated phosphorylation and ubiquitination of Miro1 and its subsequent proteasomal degradation also leads to arrest of mitochondrial transport, allowing lysosomal degradation of dysfunctional mitochondria. **(B)** Cells expressing PD-associated mutations of α-synuclein or PINK1 failed to remove Miro1 from the surface of impaired mitochondria, causing dysregulation of mitochondrial transport and delayed mitophagy. This figure was created using elements from Servier Medical Art, licensed under a Creative Common Attribution 3.0 Generic License (www.smart.servier.com).

From the molecular point of view, regulation of mitochondrial transport by mammalian Miro1 and Miro2 proteins occurs through the formation of a complex with the trafficking kinesin-binding proteins 1 and 2, so-called TRAK1 and TRAK2 (2, 115, 116). In particular, the building of the Miro/TRAK1/2 complex on the mitochondrial surface leads to the recruitment of the motor proteins kinesin and dynein for anterograde and retrograde transport, respectively (Figure 6A) (3, 117, 118).

However, the involvement of TRAK1 and TRAK2 differentially regulates mitochondrial transport. TRAK1 predominantly facilitates anterograde and retrograde movement in axons via interaction with kinesin or dynein, while TRAK2 is mostly found in dendrites binding to dynein and thus supporting retrograde movement (41, 119).

Mitochondrial transport is regulated by cytosolic calcium levels via the calcium sensor Miro1, and in 2009, two independent studies proposed different mechanisms of how calcium binding to Miro1 regulates mitochondrial transport. MacAskill et al. showed that Kif5 directly binds to Miro1 *in vitro* (93). Upon elevation of calcium levels, the EF-hand domains of Miro1 bind calcium, inducing a conformational shift and a decoupling of Miro1 and Kif5, as shown by co-immunoprecipitation in rat brain samples. Thus, the mitochondrial transport machinery is disassembled in order to derail mitochondria from the cytoskeleton (93).

In contrast, Wang and Schwarz found that kinesin is binding to Miro indirectly via Milton (*Drosophila* homolog of TRAK1/2) (3). Elevation of cytosolic calcium levels and the subsequent calcium binding to Miro allows a direct interaction of Kif5 with Miro, thereby detaching the whole transport machinery complex from the cytoskeleton and stopping mitochondrial transport in HEK cells and rat hippocampal cells. Hence, Kif5 was associated with the Miro/Milton complex on both moving and stationary mitochondria (3). This mechanism of calcium-dependent regulation of transport enables the fine-tuned arrest of mitochondria at sites of high-energy demand and cytosolic calcium levels, i.e., synapses (Figure 6A) (42, 103, 104).

Dysfunction of Miro1 causes alterations in mitochondrial transport. In rat hippocampal neurons, overexpression of Miro1 caused an increased recruitment of TRAK2 to the mitochondria, increasing the number of organelles transported to neuronal processes, while disruption of the Miro1-binding domain of TRAK2 led to mitochondrial transport arrest, showing a significant decrease in mitochondrial number in neuronal processes (93).

Miro1 is also known to interact with other PD-related proteins influencing mitochondrial transport in neurons. For example, wild-type LRRK2 and α-synuclein proteins were shown to bind to Miro1 on moving mitochondria, creating a complex that regulates the removal of Miro1 from the OMM and, therefore, promoting the detachment of mitochondria from the transport machinery (11, 12). Interestingly, the G2019S PD-associated mutation in LRRK2 disturbed this complex, inhibiting Miro1 removal from the transport machinery, thus delaying mitochondrial arrest in PD patient-derived fibroblasts and neurons (11). Moreover, the overexpression of wild-type α-synuclein and/or the PD-associated A53T mutation led to the stabilization of the α-synuclein-Miro1 complex in PD flies and human iPSC-derived neurons, preventing mitochondria to detach from the transport machinery and subsequently leading to a delayed mitochondrial arrest (Figure 6B) (12).

Furthermore, the PD-related protein PINK1 was also identified as an interaction partner of Miro1 in human neuroblastoma cells and primary fly neurons for the trafficking of mitochondria, and the loss of PINK1 led to aberrations in mitochondrial morphology and dynamics (7, 65). In addition, PD-associated PINK1 deletions promoted the movement of mitochondria via the stabilization of dMiro (Figure 6B), consequently leading to synaptic overgrowth and death of *Drosophila* dopaminergic neurons (21). Liu et al. showed in *Drosophila* muscle and dopaminergic neurons that downregulating dMiro could rescue mitochondrial transport and distribution defects observed in mutant PINK1 flies, whereas overexpressing dMiro alone led to mitochondrial enlargement and dopaminergic neuronal death (9).

### Intercellular Mitochondrial Transfer

Converging evidence supports the notion that mitochondria can be transferred between mammalian cells, in order to replace damaged organelles and prevent death of the recipient cell. There are different approaches to analyze the transfer of mitochondria between cells in co-cultures. One way is to label mitochondria of donor cells and recipient cells in red or green, respectively, allowing the detection of cells with mixed mitochondria after co-culture (120). Another approach is to label only the mitochondria from the donor cells in order to detect their transfer into recipient cells labeled with GFP, phalloidin, or cell tracker dyes (121–124).

Since the first observation of this phenomenon in human stem cells (125), intercellular transfer of mitochondria was also noticed between healthy and cancer cells (126–128) and, more relevant to neurodegeneration, between astrocytes and neurons. In particular, by using co-culture experiments, Hayakawa et al. demonstrated that transfer of mitochondria from astrocytes to neurons improved mitochondrial function of the latter, resulting in increased neuronal recovery and survival after stroke (129). The molecular mechanisms ensuring intercellular mitochondrial transfer between neuronal cell types have not been fully elucidated yet; either the selective formation of tunneling nanotubes (TNT), the establishment of gap junctions, or the release of extracellular microvesicles containing mitochondria have been observed in different non-neuronal models (126, 128, 130, 131).

Despite the fact that the last stage of the transfer is still being debated, it is widely accepted that intercellular mitochondria donation requires a fully functional mitochondrial transport machinery. In light of its fundamental role in regulating mitochondrial movement along microtubules, a strong body of evidence indicates that Miro1 also plays a key role in mitochondrial transfer between different cell types, including non-neuronal cells (120–123). For instance, inhibition of mitochondrial complex I activity by rotenone treatment significantly decreased Miro1 protein levels in mesenchymal stem cells (MSCs), leading to impaired transfer of mRFP-labeled mitochondria to recipient primary mouse epithelial cells containing mGFP-labeled mitochondria. Consequently, Miro1-depleted MSCs displayed reduced donor activity compared to control cells, a phenotype specifically linked to impaired mitochondrial movement along microtubules (120).

Importantly, Gao et al. recently demonstrated that Miro1, as well as Miro2, participates in the transfer of mitochondria between brain cells, as displayed by a reduced mitochondrial transfer efficiency from neurons expressing mito-DsRed to GFP-labeled astrocytes upon shRNA-mediated Miro1 or Miro2 downregulation. Conversely, mitochondrial transfer increased when Miro1 or Miro2 were ectopically expressed (124).

Since mitochondrial transfer is activated by loss of respiratory function in recipient cells, the presence of functional Miro1 in donor cells is crucial to enhance mitochondrial transfer capacity and rescue mitochondrial dysfunction in injured cells. This mechanism is extremely important for many neurodegenerative diseases including PD, as it may represent a key neuroprotective approach in stem cell-based regenerative medicine. At the same time, the transfer of damaged mitochondria between different cell types may also trigger the spread of PD pathology to other brain regions and therefore needs to be taken into account for the design of targeted therapies (132).

### Peroxisomal Transport

Until recently, Miro1 was described as an entirely mitochondrial protein (27, 28, 133). However, in 2017 Costello et al. revealed that Miro1 was also localized to peroxisomes in COS-7 cells (22). The peroxisomal receptor/chaperone PEX19 was found to be necessary for the integration of Miro1 into the peroxisomal membrane (23). Recent findings suggest that the N-terminal GTPase domain regulates the direct interaction of the transmembrane domain of Miro with Pex19 (134).

In 2018, Okumoto et al. showed that the localization of Miro1 to mitochondria or peroxisomes depends on the alternative splicing of exons 19 and 20. The resulting different insertions between the C-terminal GTPase domain and the transmembrane domain determine the organelle-targeting specificity of Miro1. Variant-1 of Miro1 does not contain either the exon 19 or the exon 20 and localizes exclusively to mitochondria. Variant-3 contains exon 20 and is likewise found only on mitochondria. In contrast, variant-2 contains exon 19 and localizes partially to peroxisomes, whereas variant-4 containing both exons 19 and 20 is localized mostly to peroxisomes and to a minor extent to mitochondria (24).

In human cancer cells, Miro1 variants−1 and−2 were predominantly expressed, while variants−3 and−4 showed low expression levels of 10% compared to variants−1 and−2 (24). However, in contrast to this study, Covill-Cooke et al. recently showed that Miro1 variants lacking exon 19 as well as Miro2 are able to localize to peroxisomes in MEFs (134).

Initially, the study of Castro et al. suggested the main function of Miro1 in the regulation of peroxisomal transport and transport-dependent peroxisomal proliferation in fibroblasts (23). Nevertheless, the role of Miro in peroxisomal movement was questioned later. The knockout of Miro1 or Miro2 or a double knockout of both proteins did not reveal any effect on long-range microtubule-dependent peroxisome transport in MEFs (134). Interestingly, knockout of Miro2 revealed a significant reduction in median net displacement of peroxisomes in MEFs. This short-range peroxisomal movement was independent of the integrity of the actin and microtubule cytoskeleton but followed the oscillating movements of the ER, suggesting that Miro might regulate short-range peroxisome transport via the interaction with the ER (134).

While the results of the study by Covill-Cooke suggest that the main function of Miro at peroxisomes is independent of transport, their study demonstrated a major impact of Miro on peroxisome size and number. Double knockout of Miro1 and Miro2 in MEFs caused a significant reduction in peroxisome size, accompanied by increase in peroxisome number (134). This phenotype is likely caused by an increased interaction of the fission proteins Drp1 and Fis1 at peroxisomes, indicating that Miro proteins regulate Fis1-/Drp1-dependent fission not only of mitochondria (135) but also of peroxisomes (134).

Of note, the single knockout of Miro1 or Miro2 had no effect on peroxisome size or number, while overexpression of Miro1, but not Miro2, caused an increase in peroxisome size. This result suggests that peroxisome morphology is mainly regulated by Miro1, and Miro2 has the ability to compensate for Miro1 impairment in peroxisomes (134). This is interesting because other studies demonstrated that Miro2 was not able to compensate for the lack of Miro1 on mitochondrial level in murine brains (10, 136). Future investigations will be necessary to uncover the differential functions of Miro1 and Miro2 in mitochondria and peroxisomes and their impact in neurodegeneration. Peroxisomes are critically involved in lipid metabolism and defense against ROS (137, 138). The physical link to mitochondria and the ER is important for peroxisomal proliferation and function (139, 140). Given the crucial roles of Miro1 at mitochondria and MERCs, further investigations are needed to elucidate Miro1 functions outside of mitochondria.

To date, peroxisomal dysfunction in the pathogenesis of neurodegenerative diseases like PD is not well understood. A previous study showed a reduction in plasmalogen levels in blood plasma of PD patients (25). Plasmalogens are phospholipids synthesized in peroxisomes, which are involved in the defense against ROS. Interestingly, mice deficient in the peroxisomal proteins Pex2, Pex5, or Pex13 showed an elevation of α-synuclein oligomers and α-synuclein phosphorylation in brain tissue. The observed α-synuclein aggregation correlated with changes of peroxisomal lipid synthesis instead of being associated with mitochondrial dysfunction or oxidative stress (26).

The calcium handling function of peroxisomes is largely unknown. Previous studies showed that increased cytosolic calcium concentrations lead to an elevation of peroxisome calcium levels, suggesting that peroxisomes might play a role in calcium homeostasis (141). The newly identified role of Miro1 as adaptor for peroxisomal transport, together with the known function of Miro1 in calcium homeostasis, raises the question whether Miro1 is also involved in peroxisomal calcium handling. Furthermore, it remains to be investigated how calcium transients regulate Miro1-mediated peroxisomal transport and distribution and how this would influence peroxisomal function in the healthy state and in the context of PD.

## Outlook

The emerging role of Miro GTPases in brain health and disease provides unique opportunities for a better understanding of neuronal homeostasis and indicates these proteins as potential therapeutic targets and entry-points for precision medicine. Especially from the perspective of neurodegeneration, the roles of Miro1 as an adaptor for mitochondrial transport and as a PINK1/Parkin-mediated mitophagy substrate are of high relevance in the context of brain disorders, in particular for PD. In this review, we showed that Miro1 is not only a crucial component of the mitochondrial transport machinery and mitochondrial quality control, but it is also an important regulator of mitochondrial and cytosolic calcium homeostasis, mitochondria and ER interface, and peroxisomal dynamics. Based on a variety of *in vitro, ex vivo*, and *in vivo* studies performed in yeast, animal, and human models, Miro GTPases stand no longer as exclusive mitochondrial proteins, but their recently discovered key functions further extend their physiological role to other organelles and cellular compartments.

Based on genetic studies, a direct link of Miro1 to neurodegeneration in PD was established. Together with the fact that Miro1 physically and functionally interacts with a number of PD-related proteins, Miro1 has recently been proposed both as a molecular signature in PD and as a therapeutic target, which could be used as a biomarker for the diagnosis and treatment of PD (66). Hsieh et al. were able to rescue impaired mitophagy and neuronal cell death by pharmacologically removing excess Miro1. Treatment with the compound called “Miro1 reducer” in combination with CCCP lead to Miro1 degradation and induction of mitophagy in fibroblasts derived from PD patients. Additionally, iPSC-derived neurons from PD patients displayed significant death after induction of oxidative stress via antimycin, while control neurons did not show cell death under these conditions. Remarkably, treatment with the “Miro1 reducer” rescued iPSC-derived neurons from PD patients under antimycin stress, thereby demonstrating that the removal of excess Miro1 is neuroprotective (66).

Another possible approach for pharmacological intervention was demonstrated by Lee et al. in 2016. This study revealed that the recruitment of Miro1 to MERCs depends on the phosphorylation of the N-terminal GTPase domain by Polo kinase, thereby critically regulating mitochondrial calcium uptake and mitochondrial energy production (84). Specific inhibition of Polo kinase with BI2536 reduced the localization of Miro1 to MERCs and also caused a destabilization of MERCs (84). This finding is especially interesting in the light of increased numbers of MERCs and enhanced localization of Miro1 to MERCs observed in iPSC-derived Miro1-R272Q neurons (67). Thus, pharmacologically targeting regulators of Miro1 function such as Polo kinase offers another promising approach for personalized medicine but also bears the risk of unwanted side effects.

Future studies should focus on the impact of Miro1 on neuronal homeostasis, and the establishment of screening campaigns on cellular phenotypes in patient-based cellular models should be performed to rescue impaired Miro1 function. Identified compounds may be applicable to patients beyond monogenic PD, as impaired Miro1 function was also identified in sporadic PD (11, 12, 66). Further applications beyond PD may be also considered, as functional associations between Miro1 and key proteins causative of other neurodegenerative diseases, such as Alzheimer's disease (142, 143), amyotrophic lateral sclerosis (144, 145), and Charcot–Marie–Tooth disease (146), were discovered during the past years.

## Author Contributions

DG and RK designed and organized the structure of the review. DG, CB-E, AC, and GA developed the writing of the original review draft. DG, CB-E, GA, and RK performed the revision and editing of the original draft. DG, CB-E, AC, and GA developed all the literature research for the writing of this review. Finally, CB-E performed the figures contained in the review.

## Conflict of Interest

RK serves as Editorial Board Member of the European Journal of Clinical Investigation, the Journal of Parkinsonism and Related Disorders and the Journal of Neural Transmission. RK has received research grants from Fonds National de Recherche de Luxembourg (FNR) as Coordinator of the National Centre for Excellence in Research on Parkinson's disease (NCER-PD), Coordinator of the Study on COvid-19 National survey for assessing VIral spread by Non-affected CarriErs (CON-VINCE). RK received as well speaker's honoraria and/or travel grants from Abbvie, Zambon, and Medtronic and he participated as PI or site-PI for industry sponsored clinical trials without receiving honoraria. The remaining authors declare that the research was conducted in the absence of any commercial or financial relationships that could be construed as a potential conflict of interest. The handling editor declared a past collaboration with the authors.
